# Multi-modal cross-linguistic perception of Mandarin tones in clear speech

**DOI:** 10.3389/fnhum.2023.1247811

**Published:** 2023-09-27

**Authors:** Yuyu Zeng, Keith K. W. Leung, Allard Jongman, Joan A. Sereno, Yue Wang

**Affiliations:** ^1^Department of Linguistics, University of Kansas, Lawrence, KS, United States; ^2^Department of Linguistics, Simon Fraser University, Burnaby, BC, Canada

**Keywords:** multi-modal, audio-visual, clear speech, Mandarin tone, intelligibility, cross-linguistic

## Abstract

Clearly enunciated speech (relative to conversational, plain speech) involves articulatory and acoustic modifications that enhance auditory–visual (AV) segmental intelligibility. However, little research has explored clear-speech effects on the perception of suprasegmental properties such as lexical tone, particularly involving visual (facial) perception. Since tone production does not primarily rely on vocal tract configurations, tones may be less visually distinctive. Questions thus arise as to whether clear speech can enhance visual tone intelligibility, and if so, whether any intelligibility gain can be attributable to tone-specific category-enhancing (code-based) clear-speech cues or tone-general saliency-enhancing (signal-based) cues. The present study addresses these questions by examining the identification of clear and plain Mandarin tones with visual-only, auditory-only, and AV input modalities by native (Mandarin) and nonnative (English) perceivers. Results show that code-based visual and acoustic clear tone modifications, although limited, affect both native and nonnative intelligibility, with category-enhancing cues increasing intelligibility and category-blurring cues decreasing intelligibility. In contrast, signal-based cues, which are extensively available, do not benefit native intelligibility, although they contribute to nonnative intelligibility gain. These findings demonstrate that linguistically relevant visual tonal cues are existent. In clear speech, such tone category-enhancing cues are incorporated with saliency-enhancing cues across AV modalities for intelligibility improvements.

## 1. Introduction

We experience different speech styles in face-to-face speech communication. In adverse listening conditions or when interacting with hearing-impaired and nonnative perceivers, speakers often alter their speech productions using a clarified, hyper-articulated speech style. As compared to plain, conversational speech, clear speech involves more extreme articulatory configurations and acoustic properties (e.g., Moon and Lindblom, 1994; Gagné et al., 2002; Ferguson and Kewley-Port, 2007; Maniwa et al., 2009; Kim and Davis, 2014). Such modifications can enhance intelligibility of consonants and vowels as perceivers make use of clear-speech cues from the speaker’s face as well as voice (Helfer, 1997; Ferguson and Kewley-Port, 2002; Krause and Braida, 2002; Maniwa et al., 2008; Kim et al., 2011). While substantial research focuses on clear-speech perception at the segmental level, little attention has been paid to prosody, such as lexical tone, particularly in visual perception. This is presumably because production of prosody, whose primary acoustic correlate is fundamental frequency (F0, perceived as pitch), does not rely as much on vocal tract configurations and may be less visually distinctive.

However, there is evidence that head, neck, and eyebrow movements that are typically not articulatorily relevant in segmental production may correlate with production of pitch-related variations such as tones (Burnham et al., 2001a; Chen and Massaro, 2008; Smith and Burnham, 2012). Questions thus arise as to whether such visual cues are linguistically meaningful cues to characterize tonal category distinctions or are general emphatic cues to strengthen signal saliency, particularly in clear speech. In perception, these issues raise the question of how visual cues are utilized to distinguish tonal categories when enhanced in clear speech. The present study tackles these questions by examining auditory–visual (AV) perception of Mandarin Chinese (Mandarin henceforth) tones in clear relative to plain speech by native and nonnative (English) perceivers of Mandarin.

### 1.1. Background

#### 1.1.1. Principles of clear speech

Clear-speech principles can be situated within the framework of the hyper- and hypo-articulation H & H theory (Lindblom, 1990), in terms of cue enhancement and maintenance of category distinctiveness. Specifically, clear speech has been claimed to arise from two levels of modifications: signal-based and code-based (Bradlow and Bent, 2002; Zhao and Jurafsky, 2009). On the one hand, speakers could engage in global-level, signal-based modifications that strengthen general sound saliency, for example, with greater mouth opening or increased fundamental frequency during hyper-articulation. Such modifications would be universal across speech sounds and languages, and thus may not be beneficial in the perception of specific speech sounds. On the other hand, speakers also engage phoneme-specific, code-based modifications that could augment contrasts among sound categories in a language, for example, with greater lip protrusion for rounded vowels and greater jaw lowering for low vowels as well as corresponding changes in acoustic (spectral) features (Tang et al., 2015; Leung et al., 2016; Redmon et al., 2020).

Clear-speech modifications must be able to retain such code-based phoneme-specific cues and keep any signal-based hyper-articulation within the intended category so that phonemic categorical distinctions can be maintained. This also implies that clear-speech effects may not always be beneficial, as excessively exaggerated speech resulting in overlap of phonemic categories, or attention to incorrect auditory or visual cues, may inhibit intelligibility (Kirchhoff and Schimmel, 2005). Thus, effective clear-speech modifications must involve coordination of signal- and code-based strategies to enhance as well as preserve phonemic distinctions (Moon and Lindblom, 1994; Ohala, 1994; Smiljanić and Bradlow, 2009). This may be more challenging in cases where signal-based articulatory cues such as head and eyebrow movements or acoustic cues such as F0 also serve code-based functions, as in the case of lexical tone. As such, lexical tone provides an exemplary platform for testing the clear-speech principles with respect to the extent to which signal- and code-based clear-speech tonal cues benefit intelligibility.

#### 1.1.2. Tone perception and visual cues

Unlike segments, lexical tones primarily manifest as pitch changes, which are triggered by glottal and sub-glottal activities independent of vocal tract configurations (Lehiste, 1970; Howie, 1976; Yip, 2002). Thus, it is unclear whether visual articulatory movements in tone production can provide useful cues to perception. Indeed, research on native AV tone perception in Cantonese and Mandarin has shown that performance in the AV mode is not better than in the auditory-only (AO) mode, indicating that perceivers do not additionally benefit from visual information over that provided by the auditory signal (Burnham et al., 2001a; Mixdorff et al., 2005), in contrast with the common findings of a visual benefit as well as an efficient AV integration for segments (Hessler et al., 2010, 2013). However, native perceivers’ better-than-chance performance in visual-only (VO) perception suggests that visual cues to tone may be present (Burnham et al., 2001a; Mixdorff et al., 2005). Further research has shown that, similar to the observation for segmental distinctions (Sumby and Pollack, 1954), visual tonal information may become more prominent in situations where auditory information is degraded and more difficult to access, such as in the presence of background noise or with a hearing impairment. For example, for Mandarin and Thai tones, while there was no difference in native perceivers’ identification in the AV and the AO modes, an advantage for the AV mode over the AO mode became apparent when the same stimuli were presented in babble or cafeteria noise (Mixdorff et al., 2005; Burnham et al., 2015; Hannah et al., 2017; Li et al., 2022). Similarly, when the acoustic signal of Mandarin tones was degraded to resemble cochlear-implant (CI) speech, Mandarin perceivers did significantly better in the CI-simulated AV condition than in the CI-simulated AO condition (Smith and Burnham, 2012).

The existence of phoneme-specific visual information in tone is supported by the finding that not all tones benefit equally from the presence of the speaker’s face. For example, visual gain in the AV perception of both Cantonese (Burnham et al., 2001a) and Mandarin (Mixdorff et al., 2005; Hannah et al., 2017; Li et al., 2022) tones has been found for the more dynamic contour tones (e.g., the dipping tone and the falling tone in Mandarin). Smith and Burnham (2012) found that in CI speech where pitch information is not available, pairings involving the dipping tone were better discriminated and this advantage was more pronounced in the AV condition. In the VO mode, the rising-dipping tone contrast was most easily discriminated. Likewise, Burnham et al. (2015) reported for Cantonese that the dynamic rising-falling contrast was most discriminable in the VO mode. Taken together, greater visual benefits are found for more dynamic contour tones or tone pairs that are more contrastive in contour shape.

These results are consistent with findings in production that head movements are greater for tones with greater variance in pitch (Yehia et al., 2002; Munhall et al., 2004; Garg et al., 2019). As discussed earlier, although head, neck, and eyebrow movements have been shown to be associated with tone production (Burnham et al., 2001a, 2006; Chen and Massaro, 2008; Attina et al., 2010), their articulatory source is not clear. Some of the movements (e.g., neck) are believed to be physiologically motivated, due to movements of the laryngeal muscles that control the vocal folds when pitch is varied (Yehia et al., 2002; Burnham et al., 2015). However, a physiologically motivated account is less probable for head and eyebrow movements. Recent research has related certain facial movements (e.g., head, eyebrow, lip) in terms of spatial and temporal changes in distance, direction, speed, and timing to acoustic features of tonal changes in height, contour, and duration (Attina et al., 2010; Garg et al., 2019). For example, a study from our team suggests alignments between tone articulation and pitch trajectories, with downward or upward head and eyebrow movements following the dipping and rising tone trajectories respectively, lip closing movement being associated with the falling tone, and minimal movements for the level tone (Garg et al., 2019).

These results suggest that specific movements of the head, eyebrows and lips are correlated with tonal articulation, and are likely coordinated with the spatial and temporal dynamics of the production of different tones (see Wang et al., 2020, for a recent review). However, further evidence from tone perception research is needed to determine if these facial tonal cues are linguistically relevant cues to enhance tone category distinctions in perception.

#### 1.1.3. Visual tone perception by nonnative perceivers

Just as how native perceivers resort to visual speech cues when speech distinctiveness decreases in adverse listening environments, nonnative perceivers also rely on visual information as an additional channel of input for perceiving challenging nonnative sounds (e.g., Reisberg et al., 1987; De Gelder and Vroomen, 1992; Werker et al., 1992).

Research on AV tone perception has consistently revealed that nonnative perceivers across different L1 backgrounds may all benefit from visual information. With VO input, perceivers of both tonal and non-tonal L1s are able to distinguish nonnative tones, as evidenced by their better-than-chance performance in the discrimination of Cantonese tones by English (non-tonal L1) and Thai (tonal L1) perceivers with no Cantonese background (Burnham et al., 2001b). Furthermore, a comparison between nonnative perception in the AV relative to the AO mode typically reveals a significant visual gain. For example, examining the perception of Thai tones presented in noise by native perceivers of Mandarin (a tone language), Cantonese (a tone language), Swedish (a pitch accent language), and English (a non-tonal language), Burnham et al. (2015) found that performance was consistently better in the AV than in the AO mode for all language groups; the only exception being English perceivers who exhibited comparable accuracy in AV and AO modes, which was attributed to a floor effect. Likewise, Dutch perceivers’ identification of Mandarin tones was found to be better in the AV than in the AO mode (Han et al., 2019). Thus, a visual benefit was obtained for nonnative perceivers with and without prior tone experience, suggesting the contribution of universal visual information to tone perception.

In addition to these common patterns, language-specific aspects to the processing of visual cues have also been observed. Nonnative perceivers’ sensitivity to visual speech information has been found to vary under the influence of their language backgrounds, being better at attuning to the visual cues with native-language (L1) counterparts while less sensitive to those that are unfamiliar to them in the L1 (Hazan et al., 2006; Wang et al., 2008, 2009). First, in line with the finding in the segmental domain of a greater visual reliance in nonnative relative to native perception (Chen and Hazan, 2007; Wang et al., 2008, 2009), nonnative perceivers seem to rely more on visual input in tone perception. In the perception of Mandarin stimuli with incongruent audio and visual tone input, English perceivers were found to rely more on visual facial information while Mandarin perceivers relied almost exclusively on auditory information (Hannah et al., 2017). Moreover, non-tonal perceivers appear to be more sensitive to visual information than tonal perceivers. For example, English perceivers are shown to outperform native Mandarin perceivers in their discrimination of Mandarin tones in the VO mode (Smith and Burnham, 2012). They are also better than perceivers of other languages that use pitch contrastively (Cantonese, Mandarin, Swedish) in discriminating Thai tones in VO (Burnham et al., 2015). However, non-tonal perceivers’ superior performance in the VO mode does not necessarily transfer to the AV mode. Burnham et al. (2001b) showed that while Thai perceivers were better at distinguishing Cantonese tones in noise in the AV mode as compared to the AO mode, English perceivers showed no such visual enhancement. Thus, even though non-tonal English perceivers can utilize visual cues to tone and perform above chance in the VO mode, their performance is not on par with tonal perceivers in terms of integrating this information with the auditory information.

Taken together, nonnative visual tone perception demonstrates language-universal as well as language-specific aspects as a function of perceivers’ linguistic experience. Facial cues for tone are more likely used by nonnative perceivers who find themselves in a challenging nonnative communicative situation.

#### 1.1.4. Perception of hyper-articulated tone

Research has shown that acoustic cues to segmental contrasts tend to be exaggerated in clear, hyper-articulated speech (Ferguson and Kewley-Port, 2007; Maniwa et al., 2009; Leung et al., 2016). Likewise, visual segmental cues are also more pronounced in clear speech (e.g., Kim et al., 2011; Tang et al., 2015). Critically, such enhanced acoustic and articulatory information in clear, hyper-articulated speech has been found to improve segmental intelligibility (e.g., Ferguson and Kewley-Port, 2002; Maniwa et al., 2008; Kim et al., 2011). With respect to acoustic correlates of hyper-articulated tone, research has revealed strengthened F0 changes. For example, Cantonese tones produced in noise (Lombard speech) exhibits increased F0 and more dispersed F0 trajectories compared to tones produced in quiet (Zhao and Jurafsky, 2009). Similarly, tone hyper-articulation in Cantonese infant-directed relative to adult-directed speech appears to be indexed by larger F0 range and expanded area of tone triangles in the F0 onset and offset space (Xu Rattanasone et al., 2013). Moreover, there is also evidence of code-based modifications when tonal-hyperarticulation interacts with other pitch-modulated linguistic aspects. In particular, Xu and Burnham (2010) show that F0 modifications in hyper-articulated Cantonese tones and intonation appear to be modulated independently such that category distinctions among tones are not affected by exaggerated intonation. Much less is known, however, about the articulatory facial cues of hyper-articulated tones. Han et al. (2019) analyzed videos of hyper-articulated versus plain Mandarin tone productions, where four Mandarin speakers who were language teachers were instructed to use “teaching” versus “natural” speaking styles. The authors reported a greater total amount of facial movements and longer durations in clear relative to plain speech. There were also tone-specific differences, with greater horizontal movements for the high-level tone and greater vertical movements for the rising and falling tones in “teaching” than “natural” speech. However, the measures were limited to three general facial movement measures (total amount, horizontal, vertical) and were not associated with particular facial regions (e.g., eyebrows, lips) as revealed by other research (Attina et al., 2010; Garg et al., 2019). It is thus unclear whether the exaggerated facial movements observed in clear speech are associated with linguistically meaningful tonal cues identified previously.

Moreover, there is little research on the perception of clearly produced tones, particularly about whether hyper-articulated visual cues can enhance contrastivity of tonal categories and thus improve intelligibility of different tones. To our knowledge, the only study on clear-speech tone perception is Han et al. (2019). Using a between-subjects design, this study examined nonnative perception of Mandarin tones in AV and AO, in natural (plain) and teaching (clear) styles, with four groups of (mostly Dutch-L1) perceivers. Although accuracy was higher in AV than AO, there was no significant difference between the natural- and teaching-style conditions in either mode. Analysis of individual tones based on reaction time data showed that perceivers identified the rising and falling tones more quickly in clear, teaching-style than natural productions across AV and AO. The authors speculate that this is because contoured tones are hyper-articulated to a greater degree (*cf.* Kim and Davis, 2001); the lack of any effect of speech style on the level and dipping tones may be because the articulation of the level tone involves minimal movements, while the dipping tone is the easiest to distinguish in the natural style already (*cf.* Mixdorff et al., 2005; Chen and Massaro, 2008). These results indicate that for nonnative perceivers, there may be some visual cues associated with clear-speech production of tone.

### 1.2. The present study

As discussed, clear-speech principles dictate that a balance be struck between enhancing signal saliency (signal-based modifications) and maintaining phonemic category distinctions (code-based modifications) in order to achieve optimal gains in intelligibility (Moon and Lindblom, 1994; Ohala, 1994). It is therefore critical to differentiate general emphatic and phonemic categorical cues that are used in clear-speech modifications and are adopted in perception. The above review also suggests an intertwined relationship between signal- and code-based cues for lexical tone, where signal-based articulatory features (head and eyebrow movements) and acoustic features (F0) also serve code-based functions for category distinctions. Hence lexical tone represents a unique testing case to disentangle the extent to which signal- and code-based clear-speech tonal cues benefit tone intelligibility.

In the present study, we examine how clear (relative to plain) speech affects the intelligibility of Mandarin tones in AO, VO and AV modalities by native Mandarin and English perceivers. To address the unresolved issues raised by previous research, the design of this study considers a number of factors which may help induce clear-speech effects as well as extricate the cause of such effects.

First, the inclusion of a VO (along with AO and AV) modality allows us to identify the extent to which visual cues can independently serve linguistic functions for tonal category distinctions, by examining intelligibility gain in the VO mode. Moreover, a within-subjects design adopted in this study enables direct comparisons across modalities (detailed in the Method section). As a result, differences in intelligibility gain between the three modalities may reflect the relative weighting of auditory and visual clear-speech tonal cues, as code-based auditory and visual cues may carry different weight for characterizing different tones (e.g., a level tone which involves minimal movements is less pronounced visually (Garg et al., 2019), whereas a low-pitched tone is less prominent auditorily (Zhao and Jurafsky, 2009). Furthermore, the present study involves native Mandarin as well as nonnative, non-tonal-L1 (English) perceivers. Previous research was unable to determine whether clear speech could augment native Mandarin tone intelligibility due to a ceiling effect in auditory tone perception (Han et al., 2019). Based on the claim that visual tonal cues are not necessarily used until the listening conditions become challenging (Mixdorff et al., 2005), tone stimuli in the present study have also been embedded in cafeteria noise to induce a reliance on visual input as well as a reliance on enhanced auditory input in clear speech. Native perceiver results may make it possible to pinpoint code-based clear-speech cues, since natives presumably use the same cues for tonal category distinctions in both plain and clear speech. In contrast, nonnative perceivers presumably rely more on signal-based modification in clear speech. Comparisons between native and nonnative patterns will help unravel the contributions of language-universal (more likely to be signal-based) versus language-specific (more likely to be code-based) cues used in the perception of clear-speech tones.

In sum, the present study manipulates four factors: Style (Plain vs. Clear)^1^, Modality (VO, AO, and AV), L1 (native Mandarin vs. nonnative English), and Tone (T1, T2, T3, and T4 in Mandarin) to investigate the multi-modal cross-linguistic perception of Mandarin tones in clear speech. Overall, for the effects of clear speech, we predict that clear speech can improve the intelligibility of Mandarin tones in both auditory and visual domains and for both native and nonnative perceivers. The inclusion of three modalities (VO, AO, and AV) is informative for teasing apart the contribution of visual and auditory information. We also hypothesize that intelligibility gains that differ as a function of individual tones or native groups may be attributed to code-based, phoneme-specific clear-speech cues; in contrast, common patterns across tones and perceiver groups may indicate signal-based, language-universal clear-speech effects.

## 2. Methods

### 2.1. Perceivers

Twenty-seven (27) native Mandarin perceivers (17 female; aged 19–29, mean = 22.6; 24 mainland Mandarin and 3 Taiwanese Mandarin) were recruited from the undergraduate population at Simon Fraser University, Canada. All participants came to Canada after age 12 and had lived in Canada for an average of 5 years (1–14 years). Thirty-three (33) nonnative perceivers (22 female; aged 18–21, mean = 9.2), whose L1 was English and who had no prior knowledge of a tone language, were recruited from the undergraduate population at the University of Kansas, USA. All Mandarin perceivers reported normal hearing, normal or corrected-to-normal vision, and no speech or language disorder history. Two English perceivers reported a speech or language disorder, and their data were excluded from the study.

### 2.2. Stimuli

The stimuli were four Mandarin monosyllabic words with the vowel /ɤ/ paired with the four tones, meaning “graceful” (婀, ē), “goose” (鹅, é), “nauseous” (惡, ě), and “hungry” (餓, è)^2^. The sound transcriptions next to each of the characters were *Pinyin*, the official romanization system for Standard Chinese; the diacritics resemble the F0 realization of the Mandarin tones (high-level, rising, dipping, and falling). These four tones are also labeled T1, T2, T3, and T4, respectively. The production of each token was recorded in isolation in plain and clear speaking styles.

#### 2.2.1. Speakers

Six (6) native speakers of Mandarin (3 female) provided the audio-visual stimuli. The speakers (aged 23–28, mean = 24) were recruited from the undergraduate and graduate population at Simon Fraser University. These speakers came to Canada after age 12 and had lived in the country for an average of 2.5 years (2 months to 12 years). They indicated no history of speech or language impairment.

#### 2.2.2. Elicitation and recording of plain and clear stimuli

A simulated interactive computer speech recognition program developed previously elicited plain and clear speech stimuli (Maniwa et al., 2009; Tang et al., 2015). The speaker saw one of the four Mandarin tones displayed in Chinese characters and Pinyin on a computer screen. They were asked to produce the monosyllabic word naturally, as if in a casual conversation (plain style production). After each production, the computer’s identification of the production appeared on the screen. Unbeknownst to the speakers, the speech recognition program deliberately made mistakes as well as making correct guesses occasionally. In cases of mistakes, the speaker was instructed to repeat the incorrectly identified words as clearly as possible (clear style production). In total, each speaker elicited 12 plain-only productions when the computer returned a correct guess (4 tones x 3 repetitions) and 96 (48 plain-clear pairs) productions when the computer made a wrong guess (4 tones × 2 styles × 12 repetitions). In addition to these target words, two sets of tone quadruplet words in /i/ and /u/ contexts were included as fillers. The order of recording of all the stimuli was randomized to minimize potential carry-over effects. Only the target words with paired plain-clear productions were used in the present study.

Audio-video recordings were acquired in a sound-attenuated booth in the Language and Brain Lab at Simon Fraser University (SFU). Front-view videos were captured with a Canon Vixia HF30 camera at a recording rate of 29 fps. Audio recordings were made simultaneously using Sonic Foundry Sound Forge 6.4 at a 48 kHz sampling rate. A Shure KSM microphone was placed at a 45-degree angle, about 20 cm away from the speaker’s mouth. Two phonetically trained native speakers of Mandarin evaluated each audio and video stimulus to ensure accurate pronunciation and high recording quality.

#### 2.2.3. Editing of stimuli

Three sets of stimuli were created, corresponding to three modalities: visual-only (VO), audio-only (AO), and audio-visual (AV). Removing the audio track from the video recordings created the VO stimuli. The AO stimuli were excised from the audio recordings as individual word clips using Praat (Boersma and Weenink, 2021). The AV stimuli were generated by replacing the on-camera audio track with high-quality audio recordings from the microphone (i.e., the excised AO stimuli). The video recordings were manipulated with Adobe Premier Pro CC 2014. The average duration of all sound clips was 580 ms (SD = 193 ms) across styles, tones, and speakers. Silent portions were added before and after each sound clip so that all AO stimuli lasted 2 s. All VO and AV stimuli lasted 4 s to capture both mouth opening and closing.

The AO and AV stimuli were embedded in noise to: (1) induce sufficient errors such that a clear speech enhancement (relative to plain speech) is likely to emerge; (2) balance native and nonnative performance.

A pilot study helped determine the suitable signal-to-noise ratio (SNR), testing 5 native Mandarin listeners and 5 nonnative Mandarin listeners whose L1 was English. The nonnative listeners first completed a familiarization task and achieved above-chance accuracy; they did not participate in the subsequent experiment. Participants chose which word they had heard from four alternatives displayed on the screen on each trial. Following Dees et al. (2007), 5 noise levels were tested (−3, −6, −9, −12, and −15 dB). The results suggest that if the same SNR were used across participant groups, it would be either too easy for the native listeners or too challenging for the nonnative listeners. Therefore, different SNR levels were set to achieve a comparable level of error rate across groups, following similar previous studies (e.g., Gagné et al., 2002; Dees et al., 2007; Wang et al., 2008; Redmon et al., 2020). For a target error rate set at 30%, results of the pilot testing suggested a −9 dB SNR for native Mandarin listeners and −3 dB SNR for nonnative listeners.

The audio stimuli were first normalized to 65 dB in Praat. The intensity-normalized files were then embedded in one of three stretches of cafeteria noise recorded at SFU at levels of 68 dB and 74 dB, producing one set of stimuli at −9 dB SNR (for testing native listeners) and another set at −3 dB SNR (for testing nonnative listeners).

### 2.3. Procedures

The same procedures and comparable experimental settings were administered to the Mandarin and English perceivers. Paradigm (Tagliaferri, 2005) controlled the presentation of the perception experiment. Individual perceivers were tested in a sound-attenuated room. The task was four-alternative forced-choice identification.

On each trial, a stimulus was presented. Perceivers were asked to identify what they had perceived from four alternatives displayed in one of the three formats: (1) traditional characters with Mandarin phonetic symbols for Taiwanese Mandarin perceivers; (2) simplified characters with Pinyin for mainland Chinese perceivers; (3) tone labels (level, rising, dipping, falling) for nonnative English perceivers. The perceivers were encouraged to respond as quickly as possible. If a response was not made after 4 s, the trial was classified as a timeout, and a subsequent trial started.

Before the perception experiment, nonnative perceivers had a familiarization session. The task contained 16 stimuli, consisting of two repetitions of the syllable “duo” (/tuo/) with four tones by two speakers. The stimuli were presented auditorily without noise. The perceivers first listened to sample stimuli of each tone. Then, they completed a four-alternative forced-choice identification task and received feedback about the correctness of their choice on each trial. The perceivers were encouraged to repeat the familiarization session if they were not confident about the tone and label mapping. All nonnative listeners achieved above-chance accuracy after familiarization. Thirty of the 31 nonnative perceivers passed the familiarization threshold (25%) after one familiarization session. One nonnative perceiver passed the threshold after two sessions (from 25 to 50%). The average accuracy rate of the familiarization test was 67.70% (31.25 to 100%; SD = 22.31%).

The main perception test contained 432 stimuli (6 speakers * 2 styles * 3 modalities * 4 tones * 3 repetitions) and lasted about 90 min. The three blocks (VO, AO, and AV) were counterbalanced across native participants. For nonnative perceivers, the block order was either AV-AO-VO or AV-VO-AO. The presentation order within each block was randomized.

### 2.4. Statistical analysis

Mixed-effects modeling was the primary statistical modeling method. All models were fitted using the *lme4* package (Bates et al., 2022; version 1.1–30) in R (R Core Team, 2021; version 4.2.1). The reported *p*-values were provided by the *lmerTest* package (Kuznetsova et al., 2020; version 3.1–3). All mixed-effects models used restricted maximum likelihood estimation. Model selection reflected the following considerations. First, when possible (i.e., no singular fit or convergence failure), random slopes of experimental variables were included in the maximal model to avoid inflating the Type I error rate (Barr et al., 2013). Second, since Bates et al. (2018) recommended informed parsimonious models to prevent overfitting, the maximal possible model was simplified *via* model comparison, managed by the *buildmer* package (Voeten, 2021; version 2.6).

The *phia* package (Rosario-Martinez et al., 2015; version 0.2.1) was used for post-hoc comparison. The corresponding *p*-values used the correction procedure in Holm (1979). Bootstrap resampling (number of iterations = 10,000), a robust and conservative method (Berkovits et al., 2000), was also employed to aid comparisons between individual conditions. The error bars in the figures reported in the present paper represent 95% confidence intervals (CI) based on bootstrap resampling. If two conditions do not overlap in their 95% CIs, they differ significantly. In other words, when bootstrap resampling results were reported, post-hoc comparisons were unnecessary. All bootstrap resampling was managed by the *ez* package (Lawrence, 2016; version 4.4–0).

## 3. Results

### 3.1. Overall accuracy

**Figure 1:**
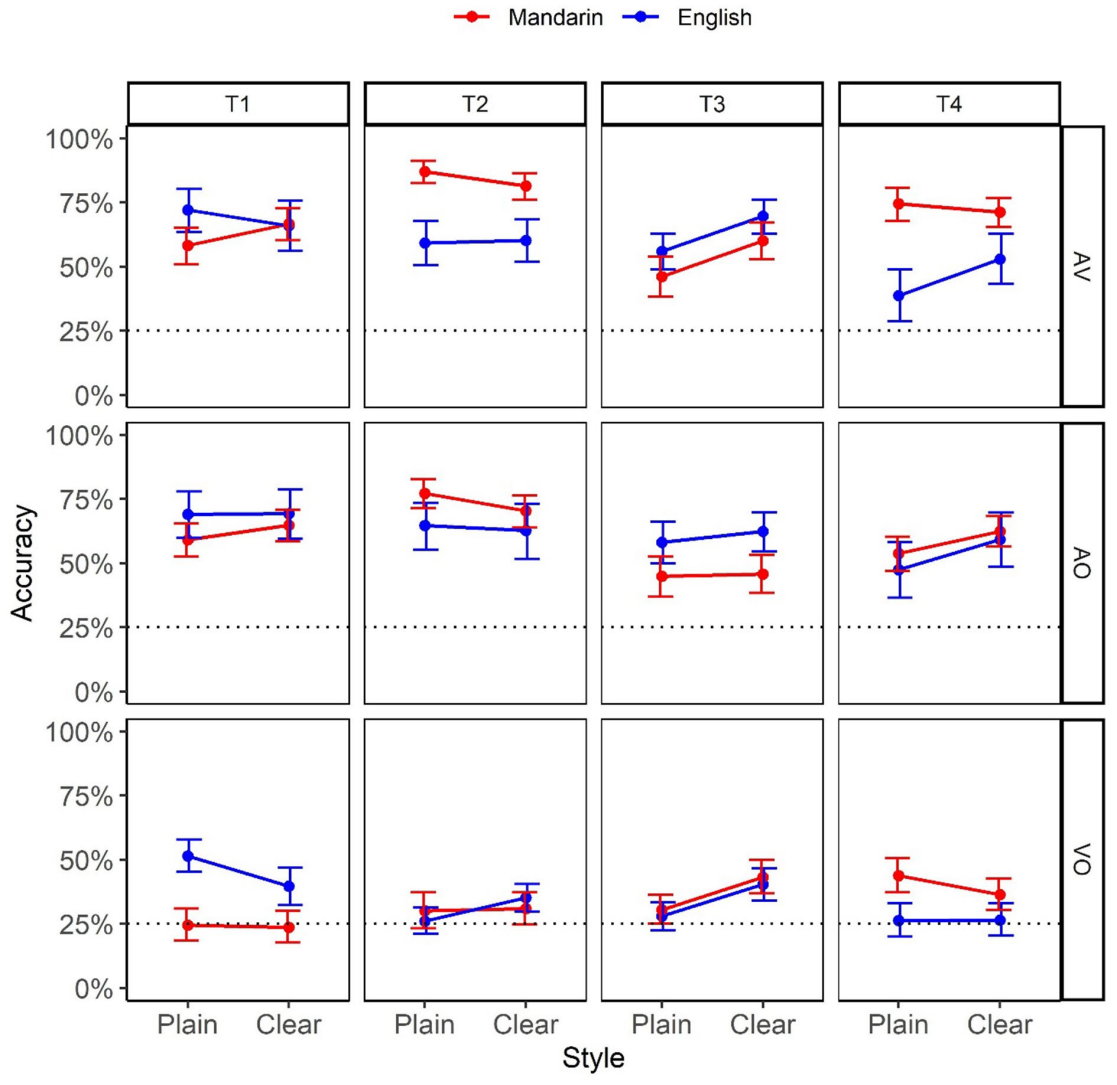
Identification accuracy as a function of Style (Plain vs. Clear), Modality (AV, AO, and VO), L1 (Mandarin vs. English), and Tone (T1, T2, T3, and T4). The numbers are based on bootstrap resampling.

Figure 1 plots tone identification accuracy as a function of Style (Plain vs. Clear), Modality (VO, AO, and AV), L1 (Mandarin vs. English), and Tone (T1, T2, T3, and T4). The horizontal dotted line represents chance level performance (25%).

A generalized linear mixed model with a binomial link function modeled the accuracy data. The dependent variable was accuracy coded in a binary format (1: correct; 0: incorrect); timeout trials without a response (0.42%) were coded as incorrect. The maximal possible model that was fed to the *buildmer* functions for model selection is as follows, using the *lme4* syntax.

Accuracy ~ Style:Modality:Tone:L1 +

(1 + Style:Modality:Tone:L1 | Participant) +

(1 + Style:Modality:Tone:L1 | Stimulus)^3^

The selected model has the following formula, whose ordering of terms indicates the contribution of each term to overall fit.

Response ~ Modality + Tone + Modality:Tone + Style + Tone:Style + L1+ Tone:L1 +Modality:L1 +Modality:Tone:L1 + Style:L1 +Tone:Style:L1 + Modality:Style + Modality:Tone:Style +.

(1 + L1+ Modality | Stimulus) + (1 + Tone + Modality | Participant)

Table 1 includes all the main effects and interaction terms in the selected model, ordered in decreasing *χ*^2^ values.

**Table 1:** Summary of fixed effects of the selected generalized linear mixed model for accuracy analysis.

Term	*χ^2^*	Degrees of freedom	*p*
Modality	167.29	2	<0.001
Modality:Tone:L1	109.56	6	<0.001
Tone:L1	34.02	3	<0.001
Modality:Tone	24.91	6	<0.001
Modality:L1	19.36	2	<0.001
Modality:Tone:Style	13.24	6	0.04
Tone	12.57	3	0.01
Tone:Style:L1	9.68	3	0.02
Tone:Style	7.93	3	0.05
Style	4.46	1	0.03
Modality:Style	0.83	2	0.66
L1	0.58	1	0.45
Modality:Style:L1	0.3	2	0.86
Style:L1	0.11	1	0.74

Significant terms are underlined, and individual terms within an interaction are joined by a colon (:). For an interaction, the order of independent variables indicates their relative contribution. For example, Modality:Tone:L1 means the effect of Modality is the largest in this interaction, with Tone being the intermediate, and L1 being the smallest.

As shown in Table 1, all manipulated variables (Style, Modality, L1, and Tone) contributed to overall fit, and were thus included in the selected model. However, only Style, Modality, and Tone were significant (with the relative effect size being Modality > Tone > Style). For the significant main effect of Style, Clear speech (54.2%, SD = 0.69)^4^ exhibited a higher perception accuracy than Plain speech (51.0%; SD = 0.69). For the main effect of Modality, post-hoc comparisons revealed that AO (60.7%, SD = 0.58) did not significantly differ from AV (63.5%, SD = 0.57; *p* = 0.20), both having a significantly higher accuracy than VO (33.6%, SD = 0.59; *p*-values <0.001). For Tone, post-hoc comparisons found T1(55.7%, SD = 0.56) and T2 (56.8%, SD = 0.57) were identified significantly better than T3 (48.9%, SD = 0.57; *p*-values <0.04), and T2 was marginally better than T4 (49.0%, SD = 0.56; *p* = 0.07); no other pairwise comparison within Tone was significant (*p*-values >0.11). Although the Mandarin perceivers (53.6%, SD = 0.50) had higher overall accuracy than the English perceivers (51.7%, SD = 0.50), the difference was not significant (*p* = 0.44), indicating the success of different SNR choices to match the two groups’ performance.

In the following section, we report results from *post hoc* analyses based on significant interactions involving Style, the focus of this study.

### 3.2. Effects of style as a function of tone, modality and L1

From the modeling results (Table 1), for the significant Tone:Style interaction (*p* = 0.05), post-hoc comparison revealed that the difference between Plain and Clear speech was only significant for T3 (*p* = 0.007; *p*-values >0.38 in other comparisons), with Clear T3 (53.8%, SD = 0.53) being more accurate than Plain T3 (44.1%, SD = 0.53). Post-hoc analyses based on the significant Tone:Style:L1 interaction (*p* = 0.02) further revealed differences in clear-speech effects as a function of L1.

Probing the presence of Style differences in Mandarin and English perceivers as a function of Tone, one marginally significant comparison was observed: T3 perceived by English participants (*p* = 0.07; *p*-values >0.25 in other comparisons); their identification of Clear T3 (58.4%, SD = 0.53) was higher than Plain T3 (48.2%, SD = 0.53). Probing the difference between Mandarin and English perceivers in individual styles as a function of Tone revealed three significant comparisons in Plain speech for the perception of T1 (Mandarin = 46.3%, SD = 0.53; English = 65.1%, SD = 0.49; *p* = 0.013), T2 (Mandarin = 63.8%, SD = 0.51; English = 50.9%, SD =0.53; *p* = 0.013), and T4 (Mandarin = 56.3%, SD = 0.52; English = 38.4%, SD = 0.49; *p* = 0.017). No other comparison was significant (*p*-values >0.33). For the significant Modality:Tone:Style interaction (*p* = 0.04), examination of the effect of Style in individual Modality as a function of Tone revealed two marginally significant comparisons: T3 in VO (*p* = 0.064) and in AV (*p* = 0.095), where Clear speech had a higher accuracy than Plain speech (41.7% vs. 29.1% in VO; 65.1% vs. 51.3% in AV). For the effect of Modality in individual styles as a function of Tone, for Plain speech, accuracy in VO was significantly lower than in AV for all four tones, and it was significantly lower than AO for T1, T2, and T3 (*p*-values <0.001) and marginally lower for T4 (*p* = 0.07). For Clear speech, VO also had a lower accuracy than AV for all four tones, and than AO for T1, T2, and T4 (*p*-values <0.001); the difference between VO and AO in T3 was not significant (*p* = 0.41). Finally, the difference between AO and AV was not significant in all possible Style * Tone combinations (*p*-values >0.99).

These results revealed a robust clear-speech advantage, particularly in the visual perception of T3. The Tone:Style:L1 and Modality:Tone:Style interactions also suggest different patterns in plain and clear conditions for the other tones and between the two L1 groups, although the multiple interaction terms may complicate meaningful interpretations of these patterns.

### 3.3. Clear speech enhancement

To facilitate interpretation, a single measure of clear-speech effect, Clear Enhancement (CE), was calculated for individual conditions using the formula^5^ below, inspired by Sommers et al. (2005):

CE = Clear – Plain/1 – Plain.

**Figure 2:**
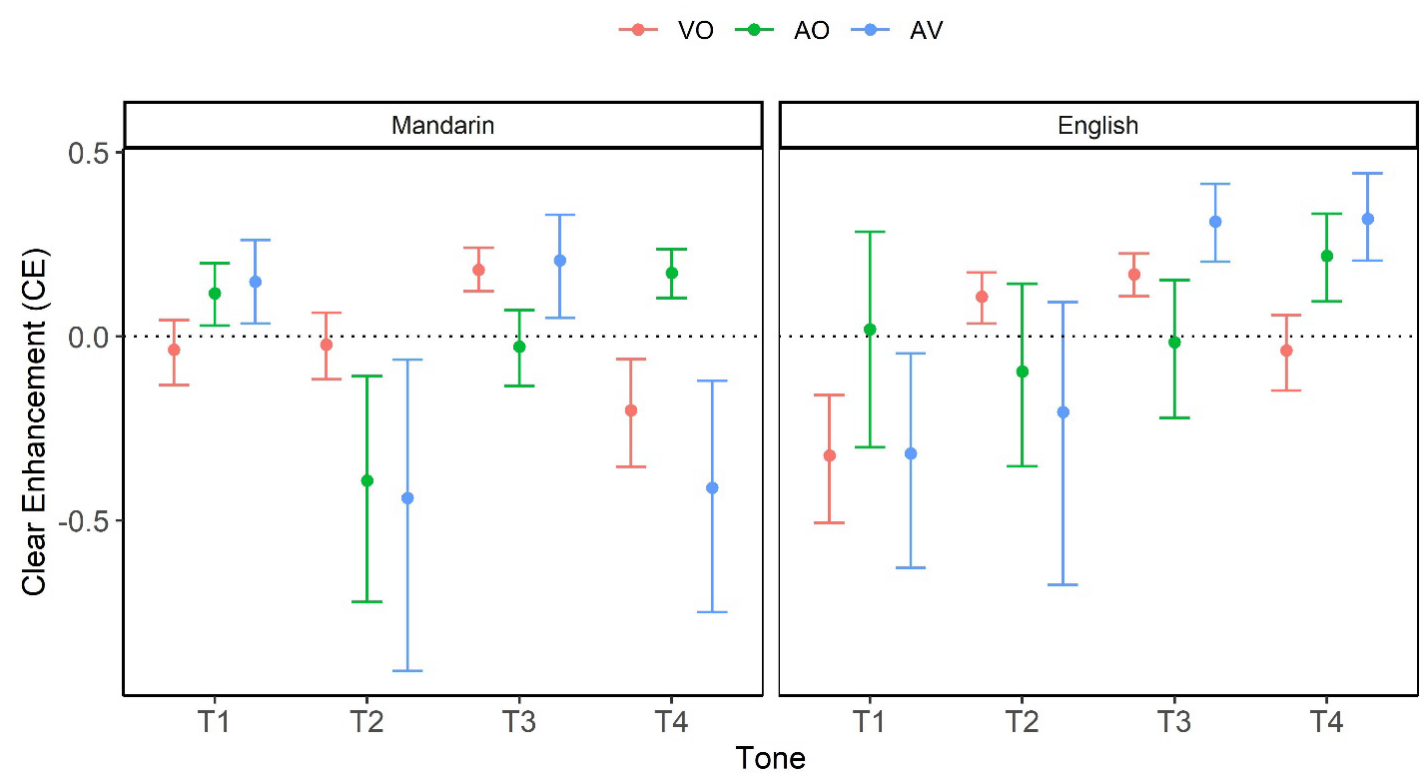
Clear enhancement (CE) as a function of Modality (VO, AO, and AV), L1 (Mandarin vs. English), and Tone (T1, T2, T3, and T4). The numbers are based on bootstrap resampling.

This formula avoids the bias inherent in a simple difference, which prevents high scorers in Plain speech from obtaining low CE. Figure 2 visualizes bootstrapped mean and 95% CI value of CE as a function of Modality, L1, and Tone. The horizontal dotted line represents no clear speech effect. Conditions above the horizontal dotted line show significant CE (Clear > Plain). As shown in Figure 2, Clear speech was perceived significantly better in the following conditions: (1) AO and AV in T1, VO and AV in T3, plus AO in T4 for Mandarin perceivers, and (2) VO in T2, VO and AV in T3, plus AO and AV in T4 for English perceivers.

Clear speech had significantly lower accuracy than Plain speech in the following conditions: (1) AO and AV in T2, plus VO and AV in T4 for Mandarin perceivers; (2) VO and AV in T1 for English perceivers.

Together, these patterns suggest a greater auditory clear-speech advantage for T1 and T4, and a greater visual clear-speech advantage for T3 and T2.

### 3.4. Visual clear-speech effects

The analyses above revealed clear speech enhancements in VO and AV conditions. Two subsequent analyses were conducted to further explore the contribution of visual information independently and integratively with auditory cues by examining (1) patterns in VO alone, and (2) visual enhancement in AV relative to AO.

#### 3.4.1. The utilization of visual tonal cues in VO

**Figure 3:**
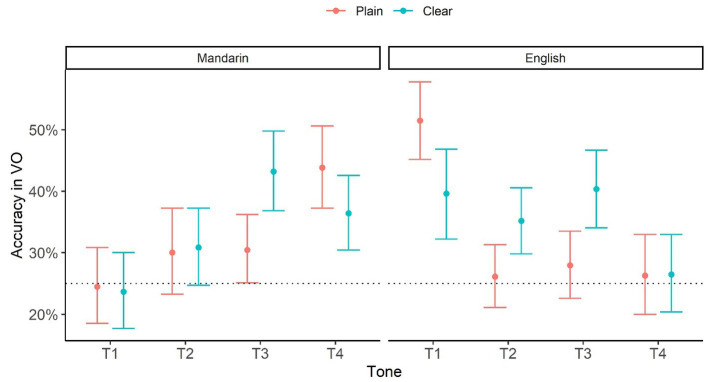
Identification accuracy in VO as a function of Style (Plain vs. Clear), L1 (Mandarin vs. English), and Tone (T1, T2, T3, and T4). The numbers are based on bootstrap resampling.

Figure 3 plots bootstrapped mean and 95% CI in VO as a function of Style, L1 and Tone based on the accuracy data. The horizontal dotted line represents chance level performance (25%).

First of all, perceivers’ performance was above chance (or marginally^6^ above chance) in the following VO conditions: Clear T2, T3, T4, and Plain T3, T4 for Mandarin perceivers; and Clear T1, T2 T3 and Plain T1 for English perceivers. Thus, both perceiver groups’ performance was above chance in more tones in the Clear than in the Plain condition, including Clear T2 and T3. Additionally, as also revealed in the modeling and CE analyses above, performance was better in Clear than Plain conditions in T3 for both groups and in T2 for English perceivers. These results show that visual information alone can provide useful cues for tone identification at greater than chance accuracy, and the effects were enhanced in clear speech, especially for T2 and T3.

#### 3.4.2. Integration of audio and visual information in clear speech

To examine how perceivers integrate visual information, Visual Enhancement (VE) was calculated for individual conditions using the next formula^7^, following Sommers et al. (2005):

VE = AV – AO/1 – AO.

**Figure 4:**
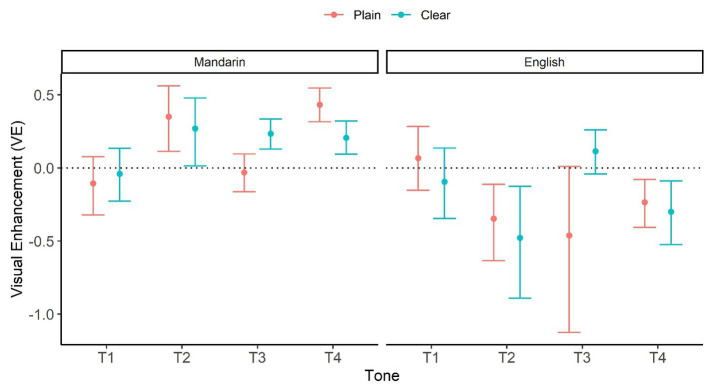
Visual enhancement (VE) as a function of Style (Plain vs. Clear), L1 (Mandarin vs. English), and Tone (T1, T2, T3, and T4). The numbers are based on bootstrap resampling.

Again, the formula avoids the bias inherent in a simple difference, which prevents high AO scorers from obtaining low VE. Figure 4 visualizes bootstrapped mean and 95% CI of VE as a function of Style, L1, and Tone. According to Figure 4, Mandarin perceivers showed significant positive VE values (AV > AO) in the following conditions: Clear T2, T3, T4, and Plain T2, T4. In contrast, English perceivers only showed marginally significant positive VE values in Clear T3. English perceivers also exhibited significant negative VE scores in T2 and T4, for both Plain and Clear speech.

Together, perceivers appeared to benefit from integrated audio and visual information more extensively (in more tones) in clear than plain speech, and native Mandarin perceivers showed this clear-speech effect more extensively than English perceivers. In addition, both Mandarin and English perceivers showed higher VE in Clear than Plain conditions for T3, consistent with the results from individual modalities.

## 4. Discussion

The present study examined the effects of clear speech on Mandarin tone intelligibility by comparing the identification of Mandarin tones in clear and plain styles in different A/V input modalities by native Mandarin and nonnative (English) perceivers. The overall results show that, across tones, modalities and native groups, clear relative to plain speech increased tone perception accuracy. This finding is in line with the segment-based findings of a clear-speech benefit across A/V and nativeness domains (Fenwick et al., 2015; Redmon et al., 2020). However, it appears to be inconsistent with the previous clear-speech tone research showing no advantage in Mandarin tone perception in teaching (clear) style compared to natural (plain) style across AO and AV conditions (Han et al., 2019).

Such seemingly discrepant patterns may be accounted for by the interactive effects of speech style with tone, modality and nativeness, since overall results across these conditions may obscure such effects. These issues are at the core of the hypotheses of the current study in terms of disentangling signal- versus code-based clear-speech tone effects, predicting that intelligibility gains that differ for individual tones and native groups would reflect code-based, phoneme-specific clear-speech effects, while common patterns across tones and native groups would indicate signal-based, language-universal clear-speech effects. Moreover, interactions of speech style and input modality would tease apart contributions of visual and auditory information in intelligibility gain, as well as identifying whether clear relative to plain tones are more visually distinctive. Understanding the extent to which clear speech benefits visual tone perception is particularly critical in unraveling the signal- versus code-based clear speech principles, given that tone is believed to be less visually distinct (Wang et al., 2020). Thus, in what follows, the results are discussed in terms of the effects of visual clear tones, A/V modalities, and L1 backgrounds.

### 4.1. Visual clear-speech effects

As stated earlier, compared to Han et al. (2019), the addition of a native Mandarin group and a VO modality allows us to pinpoint if and how well visual information can independently serve linguistic functions for tonal category distinctions and augment intelligibility in clear speech.

While there is solid evidence that visible articulatory configurations for speech can help distinguish different segments and improve intelligibility in clear speech (Helfer, 1997; Gagné et al., 2002; Hessler et al., 2010; Kim et al., 2011; Van Engen et al., 2014), the role of visual prosodic (including tonal) information remains unclear, arguably because production of prosody does not rely as much on vocal tract configurations and may thus be less visually distinguishable. As such, one plausible prediction could be that any clear-speech enhancing cues must likely be signal-based, global modifications across tones and any perceptual gain in clear speech must likely be equal across tones.

However, linking the current native Mandarin tone intelligibility results to visible articulatory correlates reveals different patterns. Specifically, the native Mandarin perception accuracy (Figure 3) and clear-speech enhancement (Figure 2) results in the VO condition consistently revealed tone-specific rather than tone-general patterns. While Tone 1 and Tone 2 did not show any critical clear-plain difference, Tone 3 exhibited a significant clear-speech improvement; Tone 4, on other hand, showed a detriment, with a decrease in performance from plain to clear conditions. These patterns have direct articulatory correlates from our antecedent study (Garg et al., 2023) examining the articulatory characteristics associated with clear-speech Mandarin tones. The articulatory analyses revealed that, while most of the 33 articulatory cues (characterizing the distance, time and speed of head, eyebrow and lip movements for tones) included in the analyses demonstrated signal-based, tone-general clear-speech modifications, only two cues appeared to be specific to individual tones. Particularly, for Tone 3, the larger and faster head-raising movement after head dipping in clear than plain speech is aligned with the dynamic nature of Tone 3 (with a falling-rising pitch contour), thus enhancing the Tone 3-specific characteristics. This is consistent with the previous findings of a greater accuracy in visual perception of Tone 3 compared to that of the other tones in Mandarin (Hannah et al., 2017; Li et al., 2022). In contrast, in Tone 4 clear speech, a larger and faster head raise occurred after head falling (that is, after the completion of Tone 4), which consequently approximated a Tone 3 movement trajectory and caused confusion. Indeed, further analysis of tone confusion patterns showed that more Tone 4 tokens were misperceived as Tone 3 in clear than plain conditions.

These results indicate an interplay between clear-speech cues in tone perception and production. Although tone-general clear-speech modifications are dominant in articulation (Garg et al., 2023), they do not contribute to much intelligibility gain. Rather, limited tone-specific modifications indeed affect intelligibility, with category-enhancing cues (for Tone 3) improving intelligibility and category-blurring cues (for Tone 4) hindering intelligibility.

### 4.2. Effects of modality

Examining the effects of input modality was critical for identifying clear-speech tone perception patterns in two aspects. First, differences in intelligibility gain between the AO and VO conditions would reflect the relative weighting of auditory and visual clear-speech tonal cues, as code-based auditory and visual cues may carry different weight for characterizing different tones, e.g., a level tone which involves minimal movements (Garg et al., 2019) may benefit less from clear-speech visually, whereas a low-pitched tone which is less prominent auditorily (Zhao and Jurafsky, 2009) may have less auditory clear-speech gain. Additionally, differences between clear-speech perception in the AV and AO conditions could unveil how well visual clear-tone information is integrated with auditory information in clear-tone perception.

Given that tone articulation is less visually distinctive, one would expect code-based acoustic clear-speech cues to benefit auditory perception. Although previous acoustic clear-speech tone analyses show both signal- and code-based clear-speech enhancing cues (Zhao and Jurafsky, 2009; Xu and Burnham, 2010; Xu Rattanasone et al., 2013), it is not clear which features benefit intelligibility. Relating the current native auditory clear tone perception results to our antecedent acoustic clear-speech tonal analyses (Tupper et al., 2021) allows a direct association of acoustic correlates with clear-speech benefits in intelligibility. From the current intelligibility results, the native Mandarin perception accuracy (Figure 3) in the AO condition revealed tone-specific instead of tone-general patterns, in that clear speech benefits auditory perception of Tone 1 and Tone 4 but not Tone 2 and Tone 3. The Tone 4 benefit can be triggered by a code-based clear-speech modification of this tone identified in Tupper et al. (2021), showing that compared to plain Tone 4, clear Tone 4 involved a steeper downward F0 slope along with a larger F0 range, which enhanced the inherent high-falling nature of this tone. It is worth noting that this Tone 4-specific clear-speech modification is the only code-based cue identified in the study; the other acoustic features (e.g., duration, mean F0, intensity) all exhibited changes across tones in clear speech. The clear-speech advantage found for Tone 1, however, could not be attributed to any tone-specific cue, since no spectral difference was found between clear and plain Tone 1 (Tupper et al., 2021).

Lastly, the AV integration results (Figure 4) revealed a visual benefit (AV > AO) for the native perceivers in the clear condition but not in the plain condition for Tone 3, indicating that the articulatory cues adopted in VO clear-speech modifications are sufficiently salient and efficiently integrated with the auditory cues to improve intelligibility in the AV condition for Tone 3. This is consistent with the VO results showing a clear-speech benefit only for Tone 3. These patterns demonstrate robust visual clear-speech information that specifically enhances the distinctiveness of Tone 3, producing an intelligibility gain in clear speech perception.

Together, the findings across modalities with native perceivers display consistent patterns of a perceptual clear-speech benefit from acoustic and visual tone modifications, in that code-based tone-specific cues, either acoustic or visual, benefit intelligibility in clear speech, whereas signal-based cues across tones, although extensively adopted in clear-speech modifications, do not result in significant perceptual gain in clear speech.

### 4.3. Effects of L1 background

Comparisons between native and nonnative patterns help unravel the contributions of language-universal (signal-based) versus language-specific (code-based) cues used in the perception of clear-speech tones. While code-based clear-speech cues, which involve language-specific properties to enhance sound category distinctions, may not benefit nonnative perceivers who cannot associate these cues with specific sound categories, signal-based cues, with enhanced saliency overall, have been shown to be beneficial to nonnative as well as native perceivers (Bradlow and Bent, 2002; Redmon et al., 2020). Given that signal-based cues are predominantly used in clear-speech tone modifications, native English perceivers in this study are expected to take advantage of these cues for intelligibility gain.

The clear-speech enhancement results (Figure 2) show that, similar to the native Mandarin patterns, English perceivers also exhibited a clear-speech advantage in identifying Tone 3 in the visual condition and in identifying Tone 4 in the auditory condition, suggesting that the code-based visual and acoustic features identified from the articulatory and acoustic analyses, respectively (Tupper et al., 2021; Garg et al., 2023), are reliable and robust clear-speech cues for Mandarin tones.

In addition to Tone 3, English perceivers revealed a clear-speech intelligibility gain for Tone 2 in the VO condition (Figure 2). Consistently, this clear-speech advantage was also observed from the accuracy analysis in VO (Figure 3), where for Tone 2 as well as for Tone3, identification accuracy was greater in clear than plain conditions, from below chance to well above chance in both cases. This additional benefit may have been derived from a greater reliance on the visual input. Previous research has firmly established that nonnative perceivers compared to native perceivers attend more to visual cues (Chen and Hazan, 2007; Wang et al., 2008, 2009; Hazan et al., 2010; Hannah et al., 2017); and English perceivers in particular, benefit more from such visual information than nonnative tone-language perceivers (Smith and Burnham, 2012; Burnham et al., 2015). The present results further suggest that attention to visual information may also enable nonnative perceivers to take advantage of enhanced (both signal-based and code-based) clear-speech cues to improve perception of nonnative contrasts.

Finally, consistent with the generally observed clear-speech advantage for visual Tone 3, the AV integration results (Figure 4) showed a greater visual gain (AV > AO) in clear than plain speech for Tone 3 for the English perceivers. However, the plain-clear difference was marginal, and the magnitude of the gain was smaller for the English than for the Mandarin perceivers. Additionally, while a visual gain was observed more extensively across tones and styles for the native Mandarin perceivers, for the English perceivers the visual contribution was not only limited (marginal for Tone 3) but also detrimental in some cases (i.e., AV < AO for Tone 3 and Tone 4 in both plain and clear conditions). These findings indicate that although English perceivers can pick up clear-speech visual cues to tones for intelligibility gains as evidenced from the VO clear speech enhancement (Figure 2) and accuracy (Figure 3) results, they are not always able to efficiently integrate the visual information with the auditory information in the AO condition. These patterns are aligned with previous findings showing English perceivers’ poorer AV integration in Cantonese and Mandarin tone perception, compared to both native and nonnative tone-language perceivers’ (Burnham et al., 2001a,b, 2015).

In sum, the native English results suggest that nonnative perceivers benefit from both signal- and code-based clear-speech cues to Mandarin tones. They particularly gain from the visual clear-speech information, although they are not proficient with AV integration of clear-speech cues.

## 5. Conclusion

The current results have established that visual information can independently serve linguistic functions for tonal category distinctions and improve intelligibility in clear speech. Moreover, tone intelligibility benefits from both acoustic and visual clear tonal cues in a complementary manner, aligned with their inherent characteristics, where acoustically prominent tones (e.g., Tone 1, Tone 4) exhibit an auditory clear-speech gain while visually prominent tones (e.g., Tone 2, Tone 3) exhibit a visual clear-speech gain. Furthermore, these tone-specific intelligibility gains have direct acoustic and visual correlates, suggesting code-based clear-speech benefits. In contrast, signal-based cues, although extensively available, contribute only to nonnative (but not native) intelligibility gain.

As a final remark, relating the current clear-speech effects on tone intelligibility to those found for segmental intelligibility (e.g., for vowels, Redmon et al., 2020) reveals differences, but ultimately, striking similarities. In terms of differences, despite the solid evidence of visual clear-speech gains for tones, the effects are smaller and less extensive compared to those for vowels. This is presumably due to the very limited code-based visual tone modifications, triggered by the lack of inherent visually distinct cues to tones, indicating that effectiveness of clear speech is determined by sound-intrinsic characteristics. On the other hand, clear-speech effects for tones and vowels bear fundamental similarities. For both, code-based clear-speech cues appear to be more effective than signal-based cues in aiding intelligibility. Critically, for both, only those code-based cues that are aligned with sound-intrinsic properties aid intelligibility while those blurring category boundaries hurt intelligibility. Thus, findings from these segmental and suprasegmental studies are in keeping with the clear-speech principles dictating a balance between enhancing signal saliency and preserving category distinctions, with code-based, category-defining cues being the most effective cues for intelligibility gains.

## Data availability statement

The raw data supporting the conclusions of this article will be made available by the authors, without undue reservation.

## Ethics statement

The studies involving humans were approved by KU Human Research Protection Program and SFU Research Ethics Board. The studies were conducted in accordance with the local legislation and institutional requirements. The participants provided their written informed consent to participate in this study.

## Author contributions

YZ: statistical analysis and writing. KL: methodology, analysis, and writing. AJ: conceptualization, review and editing, and funding. JS: conceptualization and review and editing. YW: conceptualization, writing, review and editing, project administration, and funding. All authors contributed to the article and approved the submitted version.

## Funding

This project was supported by research grants from the Social Sciences and Humanities Research Council of Canada (SSHRC Insight Grant 435–2012-1641) and the Natural Sciences and Engineering Research Council of Canada (NSERC Discovery Grant No. 2017–05978).

## Conflict of interest

The authors declare that the research was conducted in the absence of any commercial or financial relationships that could be construed as a potential conflict of interest.

## Publisher’s note

All claims expressed in this article are solely those of the authors and do not necessarily represent those of their affiliated organizations, or those of the publisher, the editors and the reviewers. Any product that may be evaluated in this article, or claim that may be made by its manufacturer, is not guaranteed or endorsed by the publisher.

## Footnotes

^1^First-letter capitalized forms of style (i.e., “Clear” and “Plain”) represent the experimental conditions in our study.

^2^The tone quadruplet with /ɤ/ context was selected because /ɤ/, as a mid-central vowel, involves neutral articulatory gestures, allowing a focus on tones without considering potential variances due to vowels, particularly in visual tone perception. This selection did not prioritize a fully balanced frequency among the four words. However, the results indicate that the participants’ responses were not biased in favor of one word over another.

^3^Stimulus refers to individual productions described in the “Elicitation and recording of plain and clear stimuli,” with specific style, tone, and speaker. The same stimulus maps to all three modalities.

^4^The numbers reported in this paper are descriptive statistics of the raw data, which can be reproduced by running the “Descriptive statistics” chunk in the shared analysis script. The values may differ slightly from the bootstrap resampling results, which are the numbers used to plot figures. However, no notable discrepancies were observed from these two analyses.

^5^When a Plain condition had a 100% accuracy, the corresponding CE score was removed from bootstrap resampling (3.07% cases), as the divisor in this case is 0 (mathematically undefined).

^6^Defined as the lower bound of 95% CI being 25% or slightly below 25%.

^7^When an AO condition had a 100% accuracy, the corresponding VE score was removed from bootstrap resampling (5.04% of all cases), as the denominator in this case is 0 (mathematically undefined).
